# Evaluation of near-infrared hyperspectral imaging for the assessment of potato processing aptitude

**DOI:** 10.3389/fnut.2022.999877

**Published:** 2022-10-17

**Authors:** Ainara López-Maestresalas, Carlos Lopez-Molina, Gil Alfonso Oliva-Lobo, Carmen Jarén, Jose Ignacio Ruiz de Galarreta, Carlos Miguel Peraza-Alemán, Silvia Arazuri

**Affiliations:** ^1^Department of Engineering, Institute on Innovation and Sustainable Development in Food Chain (IS-FOOD), Universidad Pública de Navarra (UPNA), Pamplona, Spain; ^2^Department of Statistics, Computer Science and Mathematics, Universidad Pública de Navarra (UPNA), Pamplona, Spain; ^3^Department of Plant Production, NEIKER-Basque Institute for Agricultural Research and Development, Basque Research and Technology Alliance (BRTA), Vitoria, Spain

**Keywords:** *Solanum tuberosum* L. cooking, frying as crisps, hyperspectral imaging (HSI), chemometrics, partial least squares discriminant analysis

## Abstract

The potato (*Solanum tuberosum* L.) is the world’s fifth most important staple food with high socioeconomic relevance. Several potato cultivars obtained by selection and crossbreeding are currently on the market. This diversity causes tubers to exhibit different behaviors depending on the processing to which they are subjected. Therefore, it is interesting to identify cultivars with specific characteristics that best suit consumer preferences. In this work, we present a method to classify potatoes according to their cooking or frying as crisps aptitude using NIR hyperspectral imaging (HIS) combined with a Partial Least Squares Discriminant Analysis (PLS-DA). Two classification approaches were used in this study. First, a classification model using the mean spectra of a dataset composed of 80 tubers belonging to 10 different cultivars. Then, a pixel-wise classification using all the pixels of each sample of a small subset of samples comprised of 30 tubers. Hyperspectral images were acquired using fresh-cut potato slices as sample material placed on a mobile platform of a hyperspectral system in the NIR range from 900 to 1,700 nm. After image processing, PLS-DA models were built using different pre-processing combinations. Excellent accuracy rates were obtained for the models developed using the mean spectra of all samples with 90% of tubers correctly classified in the external dataset. Pixel-wise classification models achieved lower accuracy rates between 66.62 and 71.97% in the external validation datasets. Moreover, a forward interval PLS (iPLS) method was used to build pixel-wise PLS-DA models reaching accuracies above 80 and 71% in cross-validation and external validation datasets, respectively. Best classification result was obtained using a subset of 100 wavelengths (20 intervals) with 71.86% of pixels correctly classified in the validation dataset. Classification maps were generated showing that false negative pixels were mainly located at the edges of the fresh-cut slices while false positive were principally distributed at the central pith, which has singular characteristics.

## Introduction

The potato (*Solanum tuberosum* L.) is a crop of great importance to the global economy and food security. It is the world’s fifth most important staple food, followed by rice, wheat, corn, and sugarcane. Potato production worldwide has been growing in recent decades, mainly in developing countries, reaching 370,436,581 tons in 2019 (1). This rise is largely due to the increase in both population and global per capita consumption.

In terms of potato quality, a distinction must be made between external and internal quality. The parameters that describe external quality include size, shape, color, and the presence/absence of defects. The internal quality of potatoes is defined by physicochemical parameters such as flesh color, texture, dry matter (DM) and starch content, percentage of reducing sugars (RS), susceptibility to enzymatic browning and discoloration after cooking. Several potato cultivars obtained by selection and crossbreeding are currently on the market. This diversity causes tubers to exhibit different behaviors depending on the processing to which they are subjected. Therefore, it could be convenient to identify those cultivars with specific characteristics that meet the requirements of the type of industrial processing (2, 3). The DM content for crisps industry is preferred between 22 and 24%. Higher levels result in too brittle goods while if the content is lower soft products are obtained with a higher oil retention in the frying process (4, 5). Alternatively, the optimal potato for cooking should have a lower DM content around 17 and 19% since tubers with higher levels tend to be more susceptible to bruising and disintegrate more easily when cooked (6). Regarding RS, for the crisps processing industry levels should not exceed 0.2 and 0.3% of the fresh weight to avoid both acrylamide formation during frying and products with a dark color and bitter taste that are generally rejected by consumers (4). For the cooking industry, there is no established reference limit for RS content, as cooking processes do not lead to the formation of acrylamide as temperatures are not as high as in deep-frying. However, it would be advisable not to exceed the above-mentioned limits since, regardless of their culinary suitability, these potatoes can be used for frying and thus, pose a potential risk for browning and acrylamide formation. There is a linear relationship between the content of RS in tubers and the level of browning after frying, so that the higher the former, the higher the latter (7). Likewise, some authors have found a strong correlation between the content of RS and the potential for acrylamide formation (8, 9).

Acrylamide (C_3_H_5_NO) is an organic compound formed when certain foods are cooked at temperatures usually above 120°C in low moisture conditions. It is a by-product of the Maillard reaction between free asparagine and RS. Consumption of acrylamide poses a risk to human health since it is identified as probably carcinogenic to humans. The category “potato fried products” has been pointed out as the foremost contributor to total dietary acrylamide exposure. The European Regulation (EU) 2017/2,158 established a benchmark level for acrylamide of 750 μg kg^–1^ for potato crisps (10). Therefore, the formation of this potential carcinogenic should be mitigate to the extent possible and for this, the level of RS in potato cultivars with different processing aptitudes should be known in advanced. However, as the quantity of potatoes processed worldwide is continuously increasing, their characterization and classification has become a strategic point to meet the production targets of agri-food industries. Chemical composition of tubers is generally obtained by mostly destructive and time-consuming tests. Therefore, there is a need to characterize the properties and aptitudes of tubers in a more efficient way to meet quality standards and current demand.

Non-destructive imaging-based methods are a quick and useful solution for agri-food industries, as they can provide reliable quantitative and quality information of a great range of samples including not only food but also packaging (11). In this respect, the application of hyperspectral imaging (HSI) techniques could allow the development of a fast and reliable non-destructive method to determine different characteristics of potatoes. HSI combines the advantages of traditional computer vision and spectroscopy allowing the simultaneous measurement of spatial and spectral variation of a sample (12).

For these reasons, the objective of this study is to evaluate the functionality of HSI to classify potatoes according to their frying or cooking aptitude. A field not yet addressed by this technology as far as we are concerned. To meet this target, a PLS-DA chemometric method was carried out for the classification of a dataset comprised of 80 tubers belonging to 10 different cultivars with a pre-assigned aptitude for processing as either suitable for cooking or for frying as crisps.

## Materials and methods

### Vegetal material

In this study, potato tubers of 10 different cultivars harvested in October 2016 were used.

Prior to any analysis, potatoes were characterized, and for this purpose, each tuber was identified and weighed individually. The tubers were then divided into two groups with the same number of varieties each. One group was used for the analysis of DM, starch, RS content and quality processing while the other was used for image analysis.

Information regarding these cultivars is shown in Supplementary Table 1. Five out of the 10 cultivars used were classified with industrial aptitude for cooking and the other 5 with industrial aptitude for frying as crisps.

### Analysis of dry matter, starch and reducing sugars content and quality processing

The tuber DM content was obtained by drying in an oven at 105°C for 24 h. Three samples were analyzed for each cultivar. Estimation of the RS concentration was performed by spectrophotometry based on the reduction of dinitrosalicylic acid (DNSA method) as described by Lindsay (13). A total of 0.3 g of the mixture was weighed and 1 mL of distilled water and 2 mL of dinitrosalicylic acid were added. The samples were heated at 100°C in a water bath with stirring for 10 min. Then, they were diluted with distilled water and the absorbance was measured in the UV-VIS spectrophotometer at 546 nm. The content of RS was calculated as described by Barredo (14) (Equation 1):


(1) %RS=(absorbance-0.00385)*1.07893

Specific gravity was determined by weighing a sample in air and also immersed in water, and applying a scale that gives a correlative measure of the starch content in the tuber (15).

For each potato cultivar an approximately 5 kg sample was selected for the processing quality. The tubers were stored in a cool place and kept at 8°C for 20 d after harvest. Analyses of cooked potatoes and crisps were performed. For the frying test, 3 tubers from each sample were cut into 1.5 mm thick slices with a 20 mm diameter and fried at 176 ± 5°C for 3 min in sunflower oil. The process temperature was controlled with a Hanna thermometer (Hanna Instruments, Bedfordshire, UK). The slices were drained after frying for 5 min and left at room temperature. The color score from 1 (darker) to 9 (lighter) was given to crisps according to the color chart as described by Burton (15). For the cooked potato tests, the tubers were peeled and steamed for 30 min. Each sample was assigned a value for each parameter according to the assessments of disintegration, texture firmness, mealiness, structure and flavor as described by Hassanpanah et al. (5). A panel of judges with an extensive experience from previous years was selected to determine the variations in cooked potatoes.

### Near-infrared hyperspectral imaging

Eighty tubers were used (8 potatoes per cultivar) for the image analysis. Tubers were kept refrigerated at 10°C until the subsequent analyses. Two classification groups were established for the study: cultivars with industrial aptitude for cooking, and cultivars with industrial aptitude for frying as crisps.

Hyperspectral images were acquired using fresh-cut potato slices as sample material. A thick slice (1 cm) was extracted per tuber from a transversal cut at the central part and analyzed one by one by the HSI system. A Braher slicer (Model USA280) was used to prepare the samples.

The imaging system is composed of a hyperspectral device, a mobile platform, a light source, and a computer. The hyperspectral system used consisted of a Xeva 1.7-320-100 Hz camera (Xenics, Leuven, Belgium), with a sensitive linear scanning system in the NIR range from 900 to 1,700 nm, with an InGaAs detector of 320 × 256 pixels resolution and USB connection. This camera was coupled to an ImSpector N17E spectrograph (Specim, Spectral Imaging Ltd., Oulu, Finlad) with a slit of 30 μm, and to a lens OPT-000034 (SWIR, 16 mm, f/1.4, Xenics, Leuven, Belgium) with a focal length of 16 mm. A linear actuator system (LEFS25, SMC Corporation, Tokyo, Japan) attached to a black sample holder plate was used to move the sample at constant speed through the scanning area (camera field of view).

Samples were placed in a platform 30 cm below the lens and scanned at a speed of 9 mm/s, adjusted to provide the same vertical and horizontal resolution (0.56 mm pixel^–1^). In addition, a focal length of 0.25 m and the maximum diaphragm aperture (f/1.4) were set on the lens. The images were acquired at the maximum scanning speed (100 Hz) with an integration time of 2,000 μs. All images were composed of 320 columns, 256 wavelengths (every 3.14 nm approximately) and a variable number of rows (depending on the size of the potato slice scanned).

Four 46 W halogen lamps (Lexman) emitting radiation in the infrared spectrum were used as the light source for the samples. They were positioned so that each lamp focused on one corner of the sample plate to achieve homogeneous illumination over the entire field of view. To avoid interference from external radiation from ambient light, the whole system was covered with a black opaque blanket during the image acquisition process.

A computer equipped with Xeneth 2.5 software controlled the hyperspectral acquisition system. This software allowed establishing the test parameters of the camera and controlling its operation during image acquisition.

**FIGURE 1:**
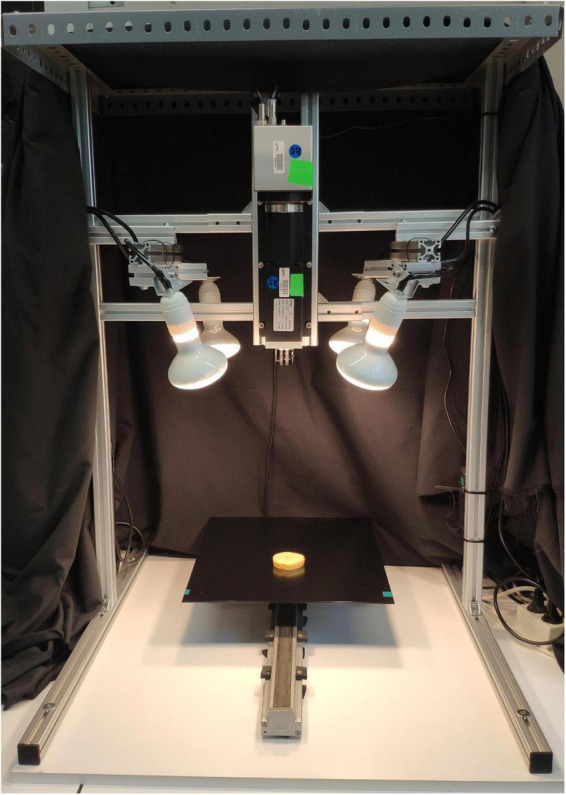
The imaging system used in this study with a sample of a potato slice placed on the platform.

Figure 1 shows the imaging system and a sample of a potato slice placed on the platform.

### Image processing

To normalize the reflectance recorded from the images with the maximum and minimum intensity captured by the receiver, images of standards with high and low reflectance coefficients, commonly called “white” and “black,” were acquired with the HSI system. The white reference was obtained by measuring a calibration tile of 600 × 50 × 10 mm with standard reflectance of 99% (Specim, Spectral Imaging Ltd., Oulu, Finland); while the dark reference was recorded by covering the lens with its opaque black cap and turning off the light source. Then, raw intensity values were converted into relative reflectance values R (x, λ) at each position x on the line and each wavelength λ of the image, using Equation (2):


(2) $$\text{R}\,\left(x,\lambda \right)=\frac{\text{I}\,\left(\text{x},\lambda \right)-{\text{I}}_{\text{D}}\,\left(\text{x},\lambda \right)}{{\text{I}}_{\text{W}}\,\left(\text{x},\lambda \right)-{\text{I}}_{\text{D}}\,\left(\text{x},\lambda \right)}$$

where I (x, λ) is the reflectance intensity of the slice potato sample and I_*D*_ (x, λ) and I_*W*_ (x, λ) are the intensity values at wavelength λ captured at the xth pixel for the dark and white references, respectively.

Then, each potato slice was segmented using the algorithm presented in Lopez-Molina et al. (16). By this procedure, a superpixel image is defined as a result of the calculation of local contrast measurements for spectral comparison based on Baddeley’s metrics. Next, the final binary region is created using Otsu thresholding algorithm and some basic morphological operations. Accordingly, with this algorithm, the potato slice was discriminated from the background.

After image segmentation, the relevant spectral data were extracted by unfolding the 3D hyperspectral array (hypercube) into a 2D data matrix of the potato pixel reflectance values at the selected wavelengths (226 bands, from 994 to 1,700 nm). The first 30 wavelengths were removed from the study due to high signal to noise ratio.

For this study, as explained further in section*-* “Partial least squares discriminant analysis (PLS-DA),” two classification approaches were used: a classification model using the mean spectra of the whole dataset (*n* = 80) and a pixel-wise classification using all the pixels of each sample of a small subset of samples (30 tubers, *n* = 143,090), with the aim of creating classification maps to discriminate the potato samples according to their industrial processing aptitude. It should be considered that a pixel-wise classification requires a fast processor as well as a large data storage due to the high number of samples used, so that sometimes it is not possible to use the whole data set in its entirety, but a smaller number of samples must be selected. In this case, 30 potato tubers, 3 tubers per cultivar, were randomly selected for the pixel-wise analysis. Moreover, for both approaches, samples were randomly divided into calibration and validation datasets. Thus, for the classification model using the mean spectra of each sample, 75% of samples were used to build the classification model (*n_*Cal*_* = 60), while the remaining 25% was used to externally validate it (*n_*Val*_* = 20). However, for the pixel-wise classification 2 tubers per cultivar were randomly selected to build the calibration group (20 samples) and the rest (1 tuber per cultivar, 10 samples) was used to validate the model. Hence, 20 hypercubes consisting of 100,166 pixels were used for model calibration and 10 hypercubes of 42,924 pixels were used for validation.

All data analysis was carried out using the PLS_Toolbox version 8.6 (Eigenvector Research Inc., Wenatchee, WA, USA) within MATLAB (release R2020b, The MathWorks, Inc., Natick, MA, USA) and additional in-house written functions.

### Spectral pre-processing

In general, spectral information, whether from conventional spectroscopy or HSI, are affected by undesirable effects such as random noise, light scattering or surface roughness in samples, among others (17). Therefore, mathematical algorithms are normally used to improve spectral data (18).

In our study, different pre-processing methods were combined to enhance the robustness of the classification models including de-noising techniques, scatter correction procedures, derivatives, and centering methods. Thus, Smoothing (SM) de-noising algorithm by the Savitzky-Golay method using a 15-point filter was applied to spectral data. In addition, Standard Normal Variate (SNV) and Multiplicative Scatter Correction (MSC) techniques were applied to correct light scattering. Although both SNV and MSC offer similar results, SNV is performed by subtracting to each spectrum its mean value and dividing by its standard deviation (19). MSC, however, requires the use of a reference spectrum to perform a linear regression of each individual spectrum onto it (20). Moreover, derivatives were used in this study to augment differences between spectra. First (1D) and second (2D) derivatives by the Savitzky-Golay algorithm using a second order polynomial and 15 window points were calculated.

Finally, scaling of data by mean-centering (MC) was performed. Using this method, the average spectrum of the dataset is subtracted from each individual spectrum.

### Chemometric methods

#### Principal component analysis

Principal Component Analysis (PCA) is an unsupervised chemometric technique commonly used prior to multivariate analysis to explore the structure of the data, as well as to identify separation trends among classes. By this method, the dimensionality of the data is highly reduced while maintaining the existing variation to some extent. For this, PCA defines new variables, Principal Components (PC), as linear combinations of the original ones and orthogonal to each other (21). The first PC covers most of the variation in the data while the second captures as much of the remaining variation as possible, and so forth.

Two PCAs were carried out in this study, 1 using the individual pixel spectra of each tuber of the calibration dataset (20 tubers, *n* = 100,166) and the other using the mean spectrum of each tuber of the whole dataset (*n* = 80). These PCAs were performed on the previously pre-processed data by means of a Savitzky-Golay SM with a 15-point window, followed by SNV and MC. Their analysis was accomplished by visually examining the scores and loadings plots.

#### Partial least squares discriminant analysis

After PCA analysis, two classification approaches were carried out using PLS-DA, one based on the mean spectra of each sample (*n* = 80) and the other performed at pixel level, namely pixel-wise classification, using only a small subset of samples (30 tubers). PLS-DA, unlike the PCA, is a supervised chemometric technique that combines linear regression by partial least squares (PLS) with discriminant analysis, through which the separation between classes might be obtained. In this way, the classification model establishes a relationship between the predictive variables X (values of the reflectance of each pixel) and the dependent variable Y (classes in the data). For this, PLS-DA creates new uncorrelated variables, Latent Variables (LV), as linear combinations of the initial ones that maximize the covariance between X and Y (22). In this study, the number of LV to be considered was established based on the model that minimized both the mean calibration and cross-validation (CV) error as suggested by Baumann et al. (23). A Venetian Blinds CV method to optimize the model and guarantee its independence within the calibration was used, with 10 divisions and 1 sample per division.

The effectiveness of the PLS-DA models was evaluated using the confusion matrix to get the accuracy, sensitivity, specificity, and class error. Confusion matrix is an ***N x N*** matrix where the elements in the diagonal are those correctly classified, i.e., the true positives (TP) and true negatives (TN), while the elements outside the diagonal are misclassified, i.e., the false positives (FP) and false negatives (FN). The sensitivity, specificity, and class error take values from 0 to 1 such as the closer to 1 the sensitivity and specificity and, the closer to 0 the error, the most accurate the classification of the samples. They are calculated as (Equations 3–6):


(3) $$\text{Accuracy}\left(\%\right)=\frac{\text{TP}+\text{TN}}{\text{TP}+\text{TN}+\text{FP}+\text{FN}}\text{x}100$$

(4) $$\text{Sensitivity}=\frac{\text{TP}}{\text{TP}+\text{FN}}$$

(5) $$\text{Specificity}=\frac{\text{TN}}{\text{TN}+\text{FP}}$$

(6) Class⁢error=1-((Sensitivity+Specificity)/2)

A suitable classification performance would present high accuracy, sensitivity, specificity, and low class error. Moreover, a classification image for the validation dataset was displayed to visualize the distribution of correctly and incorrectly classified pixels.

#### Variable selection

HSI systems generate an enormous amount of data due to the large number of wavelengths they cover. However, these data often present collinearity problems, in addition to the complexity of their handling. Therefore, it is convenient to select a few bands containing the most variability and thus, most significant information for the implementation of these HSI in automatic in-line sorting and grading systems (24). For this reason, in this study we used interval partial least squares regression (iPLS) to find the most suitable wavelengths ranges for the classification of tubers. This is a variable selection method developed by Nørgaard et al. (25) to optimize and help in the interpretation of PLS regression models. The principle of iPLS is to divide the full spectrum into smaller equidistant regions and develop PLS regression models for each of the intervals. Afterward, a comparison between the prediction performance of these local models and the full-spectrum model is made mainly considering the root mean squared error of cross-validation (RMSECV) although other parameters are also evaluated (25).

We used forward iPLS to reduce the number of variables selecting an automatic number of intervals with an interval size of either 1 or 5.

## Results and discussion

### Chemical analysis and quality processing

Table 1 gathers the DM, starch and RS content of the different cultivars used in this study in percentage of fresh weight.

**TABLE 1:** DM, starch, and RS content of potato cultivars.

Cultivar	DM (%)	Starch (%)	RS (%)
Ambition	18.56 ± 0.18	11.47 ± 0.09	0.145 ± 0.008
Laudine	18.57 ± 0.19	11.49 ± 0.11	0.116 ± 0.006
Levantina	18.63 ± 0.22	11.55 ± 0.07	0.150 ± 0.010
Madeleine	17.77 ± 0.23	10.66 ± 0.10	0.162 ± 0.012
Rudolph	18.87 ± 0.14	11.79 ± 0.12	0.076 ± 0.007
Agria	21.00 ± 0.23	13.95 ± 0.11	0.140 ± 0.012
Corsica	20.93 ± 0.15	13.91 ± 0.14	0.065 ± 0.009
Hermes	22.81 ± 0.17	15.84 ± 0.09	0.099 ± 0.010
Lady Amarilla	20.47 ± 0.21	13.44 ± 0.16	0.103 ± 0.011
Lyoness	23.14 ± 0.14	16.18 ± 0.10	0.061 ± 0.007

Values are expressed as mean ± SD.

Tables 2, 3 include the information obtained for the quality processing parameters measured for each industrial aptitude.

**TABLE 2:** Values assigned to each parameter measured for cultivars with cooking aptitude.

Cultivar	Disintegration^a^	Texture firmness^b^	Mealiness^c^	Structure^d^	Flavor^e^	Cooking performance
Ambition	C	C	A	A	A	Poor
Laudine	A	B	B	A	B	Very good
Levantina	B	B	A	B	C	Good
Madeleine	B	B	A	A	A	Good
Rudolph	B	B	A	A	C	Good

^*a*^A = none; B = light; C = moderate; D = complete.

^*b*^A = strong; B = rather strong; C = rather soft; D = soft.

^*c*^A = none; B = light; C = mealy; D = very mealy.

^*d*^A = fine; B = rather fine; C = rather coarse; D = coarse.

^*e*^A = neutral; B = moderate; C = rather strong; D = strong.

**TABLE 3:** Evaluation of frying as crisps at 176 ± 5°C.

Cultivars	Color^a^	Crisps performance
Agria	7	Good
Corsica	8	Very good
Hermes	8	Very good
Lady Amarilla	7	Good
Lyoness	7	Good

^a^1–4 = very dark brown, non-accepted; 5–6 = strong golden, accepted; 7–9 = pale golden, accepted.

It can be seen in Table 1 that all cultivars with cooking aptitude contained lower DM concentrations, between 17 to 19%. As explained in section- “Introduction,” higher levels of DM lead to a more easily disintegration. In this study, cultivar “Ambition” provided the worst disintegration and texture firmness results among the rest of the cultivars with a moderate performance and a classification as “rather soft” (Table 2). In these two categories the rest of the cultivars gave none or light disintegration and were classified as “rather strong.” Regarding the rest of the parameters, “Ambition” was identified as not mealy, with a fine structure and neutral flavor; however, the overall cooking performance of this cultivar was poor in contrast with the rest of the cultivars with good and very good cooking performances. “Laudine” was the only cultivar with a very good cooking performance due to its lack of disintegration when cooked along with its fine structure. Cultivars “Levantina” and “Rudolph” did not perform as good, probably because of their rather strong flavor. Likewise, the overall cooking performance of the cultivar “Madeleine” was good, maybe due to its light disintegration combined with an only “rather strong” texture firmness (Table 2).

DM content of cultivars with industrial aptitude for frying as crisps ranged between 20 and 23%, slightly below the preferred levels reported by Nivaa (4). However, all those 5 cultivars performed either good or very good at the evaluation of frying as crisps based on the color developed after frying at 176 ± 5°C (Table 3).

Regarding RS, the content was below 0.2% for all cultivars included in the study. In general, cultivars with industrial aptitude for cooking reported higher levels of RS excepting cultivar “Rudolph” with 0.076%. Among cultivars with frying aptitude, “Agria” provided the highest concentration of RS with 0.14%. These results are in accordance with those reported by Gallego et al. (2) for cultivars “Agria” and “Hermes” regarding DM content, while the RS contents provided were somewhat lower than the ones obtained here (1.1 g kg^–1^ and 1.4 g kg^–1^ of fresh weight for “Agria” and “Hermes,” respectively). In any case, authors confirmed the suitability of both cultivars for the frying processing industry. Hassanpanah et al. (5) while studying the cooking quality characteristics of advanced clones and potato cultivars, also found “Agria” the cultivar with the highest content of RS. However, authors reported the suitability of cultivar “Agria” for French-fry industry while here it is considered preferable for crisps industry. In a study carried out by Amrein et al. (8) to evaluate the potential for acrylamide formation in potatoes, authors obtained a content of RS for the cultivar “Hermes” very similar to the one obtained here (904 mg kg^–1^) and slightly lower for cultivar “Agria” compared to the result in this study (1,020 mg kg^–1^). They also found a strong correlation between RS and the potential for acrylamide formation for the cultivars studied, reporting levels of acrylamide of 703 and 791 μg kg^–1^ for cultivars “Agria” and “Hermes,” respectively. It should be noticed that these values of acrylamide are, respectively, below and above the benchmark level established by the European Regulation (EU) 2017/2158. Yang et al. (7) analyzed 8 different potato cultivars to determine their aptitude for processing. They also found cultivar “Agria” suitable for frying due to its DM and RS contents, similar to those obtained here. Same authors studied the influence of the frying process and potato cultivar on acrylamide formation reporting RS content of cultivar “Agria” in line with this study (26). They reported higher acrylamide levels in fried potato products when higher frying temperatures were used; however, the degree of increase was different among the cultivars studied.

### Spectral pre-processing

**FIGURE 2:**
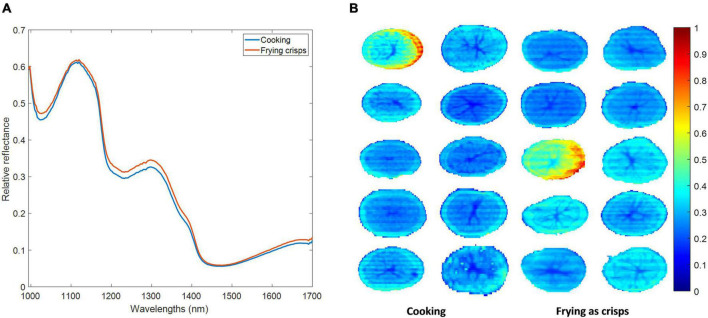
Mean reflectance spectra of all tubers belonging to cooking and frying as crisps classes **(A)** and reflectance intensity values of all pixels at 1,210 nm of cooking and frying as crisps classes included in the calibration dataset in the pixel-wise classification approach **(B)**.

Figure 2A shows the mean reflectance spectra of the 80 tubers with either cooking or frying industrial aptitude used in this study. Small differences in the magnitude of reflectance of both classes can be seen at the very beginning of the spectrum and in the 1,200–1,.400 nm where pixels from tubers with aptitude for frying as crisps showed slightly higher reflectance values than pixels from the tubers within the cooking class. Moreover, 3 major reflectance valleys are observed at around 1,015, 1,200, and 1,450 nm corresponding to absorption bands. The strong absorption band at 1,450 nm is due to O–H bond stretching and first water overtone. The absorption band at 1,200 nm corresponds to a weak water combination band and that at 1,015 nm is related to C–H stretching vibration modes in CH_3_ groups (27).

Figure 2B shows the relative reflectance of the pixels from the two classes (cooking and frying as crisps) included in the calibration dataset of the pixel-wise classification approach at a randomly selected wavelength (1,210 nm). At this wavelength reflectance values mostly ranged between 0 and 0.6 with few exceptions.

Potato tubers do not have a homogeneous distribution of components along the tuber since they present compositional gradients in radial direction from the pith to the peel (28). This was also reflected in their spectral behavior as Figure 2B shows, pixels belonging to the central pith showed the lowest reflectance values (closer to cero). As many authors have pointed out, the central pith of the potato has statistically significant lower DM content than the rest of the tissues (28), since pith tissue contains relatively few starch granules and cortical tissue is packed with them (29). This fact translates into a higher water content and, consequently, a lower spectral reflectance.

**FIGURE 3:**
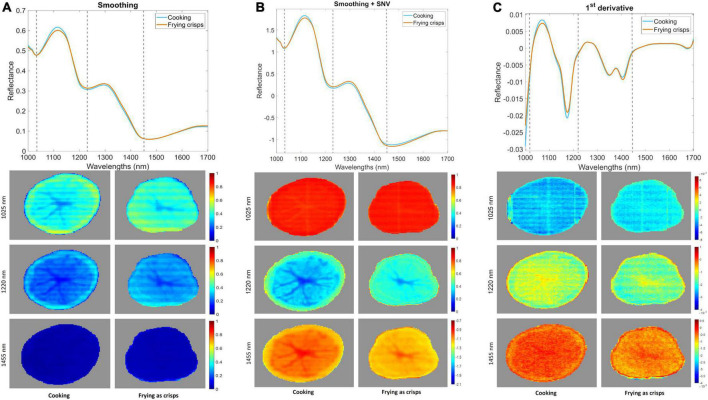
Influence in the visualization of samples of different pre-processing techniques: smoothing **(A)**, smoothing + SNV **(B)** and 1st derivative **(C)** at three specific wavelengths (1,025, 1,220, and 1,455 nm).

Figure 3 shows different spectral pre-processing methods and their influence in the visualization and possible discrimination between classes of fresh-cut potato slices in 3 different wavelengths: 1,025, 1,220, and 1,445 nm. The application of smoothing (Figure 3A) helped eliminating the spectral noise, but it was not possible to separate among cooking and frying as crisps classes. The combination of smoothing and SNV (Figure 3B) also corrected the scattering effect that is usually present in NIR radiation. Moreover, it appeared that a slight separation between classes could be accomplished at the 1,220 and 1,445 nm wavelengths. This behavior was also perceived after the application of a 1st derivative (Figure 3C), but only at 1,445 nm.

### Principal component analysis

**FIGURE 4:**
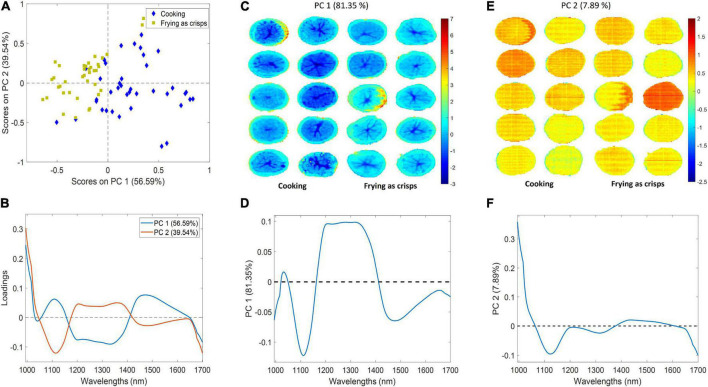
PCA models. Score **(A)** and the corresponding loading **(B)** plot from the first two PCs of the PCA using the mean spectra of all samples. Score **(C)** and corresponding loading of PC 1 **(D)**; score **(E)** and corresponding loading of PC 2 **(F)** of the PCA model using the individual pixel spectra of each tuber of the calibration dataset. Explained variance of each PC is indicated in brackets.

As mentioned before, two PCAs were performed to explore the variation of the two classes analyzed (cooking and frying as crisps) using both the pixel and mean spectra of the samples previously pre-processed by SM+SNV+MC. First, a PCA using the mean spectra of each tuber of the whole dataset (*n* = 80) was developed. In this case, PC 1 explained 56.59% of total variance while PC 2 39.54%. Figures 4A,B show, the score and loading plot of the first two PCs, respectively. According to Figure 4A it appears that PC 1 played a significant role in the separation of the classes, as all the frying as crisps samples had negative score values on PC 1 except from two samples belonging to cultivar “Lady Amarilla.” Besides, most samples belonging to the cooking class had positive score values on PC 1. According to the loadings plot (Figure 4B), potatoes with frying aptitude can be related to the wavelength range from 1,200 to 1,350 nm; while potatoes with cooking aptitude can be related to a specific band at around 1,100 nm and to the wavelength range from 1,450 to 1,700 nm.

Then, a PCA using the individual pixel spectra of 20 tubers comprising the calibration dataset (*n* = 100,166) was carried out to generate a score image plot and get an overview of the distribution of the spectral data information. The first two PCs explained 89.24% of the total variance (81.35 and 7.89%, respectively). Looking at the score surface and the corresponding loading of PC 1 (Figures 4C,D, respectively), a subtle difference could be observed between the score values of the cultivars with cooking aptitude and those with frying as crisps aptitude. The formers presented, in general, lower values (dark blue) than the pixels of samples with frying aptitude (light blue). It should be noted that the pixels from the tuber pith tissue presented the lowest score values for all cultivars in PC 1 as in concordance with the relative reflectance values shown in Figure 2B. The correspondence between the negative part of the score surface and the loading suggest that the peak located around 1,100 nm and the last wavelength range (1,450–1,700 nm) could be the main sources of difference for the two aptitudes. PC 2 scores and loadings (Figures 4E,F, respectively) did not provide any substantial information regarding the industrial aptitude of the cultivars. Moreover, some strikes are observed in Figure 4E due to the noise generated by the system.

### Partial least squares discriminant analysis

Table 4 shows the results of the 10 PLS-DA models developed using different pre-processing combinations and the mean spectra of the whole dataset. It shows the number of LV used; the percentage of variance explained; the sensitivity and specificity of each class (cooking and frying as crisps) and the overall error and accuracy obtained for each model in the CV and external validation datasets.

**TABLE 4:** PLS-DA results of classification based on the mean spectra of the CV and external validation datasets using different pre-processing combinations.

Pre-processing	#LV	Variance (%)	Class	Cross-validation (CV)				External validation			
				Sensitivity	Specificity	Error	Accuracy (%)	Sensitivity	Specificity	Error	Accuracy (%)
None	4	99.99	“C”	0.821	0.968	0.105	89.83	0.727	0.889	0.191	80
			“FC”	0.968	0.821			0.889	0.727		
MC	4	99.92	“C”	0.786	0.967	0.158	84.48	0.727	0.889	0.191	80
			“FC”	0.967	0.786			0.889	0.727		
SM+SNV+MC	4	98.69	“C”	0.889	0.968	**0.071**	**91.38**	0.818	1	**0.090**	**90**
			“FC”	0.968	0.889			1	0.818		
SM+MSC+MC	3	97.64	“C”	0.889	0.935	0.104	89.66	0.800	0.889	0.155	84.21
			“FC”	0.935	0.889			0.889	0.800		
1D+MC	3	98.15	“C”	0.750	0.966	0.142	85.96	0.818	0.889	0.146	85
			“FC”	0.966	0.750			0.889	0.818		
2D+MC	3	96.97	“C”	0.778	0.967	0.146	85.96	0.750	0.889	0.180	82.35
			“FC”	0.967	0.778			0.889	0.750		
1D+SNV+MC	3	97.46	“C”	0.828	0.931	0.120	87.93	0.881	0.931	0.146	85
			“FC”	0.931	0.828			0.931	0.881		
1D+MSC+MC	3	97.77	“C”	0.852	0.903	0.138	86.21	0.818	0.889	0.146	85
			“FC”	0.903	0.852			0.889	0.818		
2D+SNV+MC	3	94.51	“C”	0.857	0.903	0.137	87.93	0.818	0.889	0.146	85
			“FC”	0.903	0.857			0.889	0.818		
2D+MSC+MC	4	98.42	“C”	0.929	0.857	0.142	85.71	0.818	0.889	0.146	85
			“FC”	0.857	0.929			0.889	0.818		

LV, Latent variables; “C,” cooking; “FC”, Frying as crisps. Values in bold correspond to the best results obtained in terms of highest accuracy and lowest error.

High accuracy rates above 84% were obtained for all the models in CV. The highest accuracy among the models developed was obtained for the combination of SM+SNV+MC with more than 91% of samples correctly classified and an error rate of 0.071. This model was built using 4 LV explaining 98.69% of variance. Regarding sensitivity and specificity values, cultivars with industrial aptitude for frying as crisps were better classified into their class as they achieved a higher sensitivity value than the cultivars with cooking aptitude.

Very good classification results were obtained for the external validation dataset with accuracies between 80 and 90%. In this case, also the combination of SM+SNV+MC achieved the best results with 90% of samples correctly classified and the smallest error rate among the rest of the models (0.090).

### Pixel-wise classification

Table 5 shows the results of the 10 PLS-DA models developed using different pre-processing combinations in the pixel-wise classification model. It shows the number of LV used; the percentage of variance explained; the sensitivity and specificity of each class (cooking and frying as crisps) and the overall error and accuracy obtained for each model in the CV and external validation datasets.

**TABLE 5:** PLS-DA results of the pixel-wise classification of the CV and external validation datasets using different pre-processing combinations.

Pre-processing	#LV	Variance (%)	Class	Cross-validation (CV)				External validation			
				Sensitivity	Specificity	Error	Accuracy (%)	Sensitivity	Specificity	Error	Accuracy (%)
None	5	99.97	“C”	0.662	0.852	0.243	75.61	0.583	0.746	0.335	66.64
			“FC”	0.852	0.662			0.746	0.583		
MC	5	99.58	“C”	0.700	0.814	0.242	75.72	0.580	0.756	0.332	66.99
			“FC”	0.814	0.700			0.756	0.580		
SM+SNV+MC	5	94.62	“C”	0.698	0.866	**0.218**	**78.11**	0.667	0.693	**0.320**	**67.99**
			“FC”	0.866	0.698			0.693	0.667		
SM+MSC+MC	5	95.88	“C”	0.700	0.862	**0.218**	**78.05**	0.662	0.697	**0.320**	**67.97**
			“FC”	0.862	0.700			0.697	0.662		
1D+MC	7	98.71	“C”	0.681	0.862	**0.228**	**77.05**	0.640	0.732	**0.313**	**68.83**
			“FC”	0.862	0.681			0.732	0.640		
2D+MC	6	95.31	“C”	0.629	0.804	0.283	71.54	0.505	0.821	0.337	66.78
			“FC”	0.804	0.629			0.821	0.505		
1D+SNV+MC	6	95.72	“C”	0.748	0.829	**0.211**	**78.92**	0.691	0.750	**0.279**	**71.97**
			“FC”	0.829	0.748			0.750	0.691		
1D+MSC+MC	6	97.39	“C”	0.739	0.830	**0.215**	**78.39**	0.704	0.672	**0.311**	**68.76**
			“FC”	0.830	0.739			0.672	0.704		
2D+SNV+MC	7	92.71	“C”	0.769	0.714	0.245	75.48	0.692	0.660	0.323	67.62
			“FC”	0.741	0.769			0.660	0.692		
2D+MSC+MC	5	93.82	“C”	0.761	0.681	0.301	72.16	0.656	0.702	0.321	67.95
			“FC”	0.681	0.761			0.702	0.656		

LV, Latent Variables; “C,” Cooking; “FC,” Frying as crisps. Values in bold correspond to the best results obtained in terms of highest accuracy and lowest error.

Accuracy rates above 71% were obtained for all the models in CV. The highest accuracy among the models carried out was obtained for both the combination of 1D+SNV+MC and 1D+MSC+MC with very similar results, 78.92 and 78.39% with an error rate of 0.211 and 0.215, respectively. Six LVs were used to build these PLS-DA models both accounting for more than 95% of explained variance. In these 2 models, the cultivars with industrial aptitude for frying as crisps achieved higher sensitivity values than the cultivars with cooking aptitude, which means they were better classified into their group. This was true for all models except for the ones using the combination of 2D+SNV+MC and 2D+MSC+MC where potatoes suitable for cooking were better classified than cultivars suitable for frying as crisps.

Regarding the external validation, accuracies ranged between 66.64 and 71.97%, being also the model pre-processed with 1D+SNV+MC the one achieving the highest rate of correctly classified samples with the lowest error rate (0.279).

Of the 10 PLS-DA models developed, only the 5 with the best results were chosen for variable selection (values in bold). Forward iPLS was used by automatically selecting the number of intervals and establishing an interval size of either 1 or 5 depending on the pre-processing method applied. The results are shown in Table 6. The sensitivity, specificity, class error and accuracy values of the CV and external validation datasets after iPLS and different pre-processing algorithms are shown. For the CV dataset slightly better classification results were obtained with a considerably smaller number of variables. This was even more remarkable in the case of the 1D+MC pre-processed spectra where only 24 variables were used to build the classification models. Supplementary Table 2 is an extension of Table 6 including the wavelength ranges selected for each combination of pre-processing. It can be seen in Supplementary Table 2 that in the combinations of SM+SNV+MC and SM+MSC+MC the last part of the spectral range was discarded and only wavelengths up to 1,430 nm were used to build the classification models whereas in the rest of the models, the used intervals or wavelengths were distributed over the entire spectral range.

**TABLE 6:** PLS-DA results of classification of the CV and validation datasets using different pre-processing combinations and iPLS.

Pre-processing	Interval size	#V	#LV	Variance (%)	Class	Cross-validation (CV)				External validation			
						Sensitivity	Specificity	Error	Accuracy (%)	Sensitivity	Specificity	Error	Accuracy (%)
SM+SNV+MC	5	110	5	97.31	“C”	0.695	0.872	0.216	78.26	0.669	0.698	0.316	68.35
					“FC”	0.872	0.695			0.698	0.669		
SM+MSC+MC	5	105	5	97.93	“C”	0.704	0.870	0.213	78.59	0.699	0.670	0.315	68.42
					“FC”	0.870	0.704			0.670	0.699		
1D+MC	1	24	6	99.43	“C”	0.719	0.861	0.209	78.93	0.712	0.669	0.309	68.99
					“FC”	0.861	0.719			0.669	0.712		
1D+SNV+MC	5	95	7	98.61	“C”	0.771	0.833	**0.198**	**80.16**	0.767	0.626	**0.303**	**69.48**
					“FC”	0.833	0.771			0.626	0.767		
1D+MSC+MC	5	100	5	95.14	“C”	0.760	0.850	**0.194**	**80.49**	0.766	0.670	**0.281**	**71.86**
					“FC”	0.850	0.760			0.670	0.766		

V, Variables; LV, Latent Variables; “C,” Cooking; “FC,” Frying as crisps. Values in bold correspond to the best results obtained in terms of highest accuracy and lowest error.

As in the PLS-DA models using the full-spectrum, the best accuracies were obtained for the 1D+SNV+MC and 1D+MSC+MC combinations with more than 80% of correctly classified samples using 95 and 100 wavelengths (19 and 20 intervals), respectively, in the CV datasets.

Supplementary Figure 1 shows the results of the forward iPLS method obtained for the dataset pre-processed with 1D+MSC+MC. The used intervals (in green) were distributed over the entire spectral range.

**FIGURE 5:**
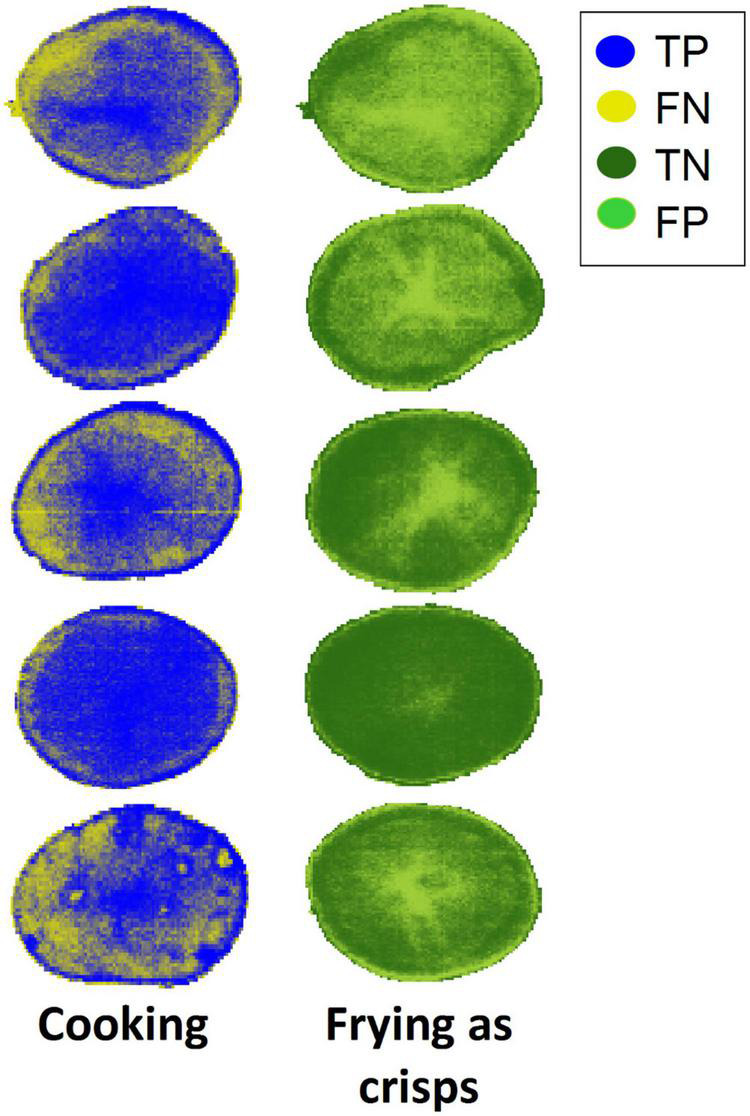
Classification maps for the validation dataset obtained by the PLS-DA model built from 1D+MSC+MC pre-processed spectra (1D+MSC+MC of Table 6) in the pixel-wise classification model.

Regarding the external validation dataset, good classification results above 68% of correctly classified pixels were obtained for the 5 PLS-DA models. The highest accuracy (71.86%) and lowest error (0.281) was obtained for the 1D+MSC+MC pre-processed spectral data. Figure 5 shows the classification maps for the10 tubers composing the validation dataset of the classification model after application of 1D+MSC+MC. For this, the matrix obtained in the PLS-DA prediction containing the estimated class assigned to each pixel needed to be folded back. In this way, the spatial distribution of the classified and misclassified pixels could be explored.

Misclassified pixels in tubers from cooking class (in yellow, false negatives) were mostly located at the edges of the slices while misclassified pixels in tubers from frying class (in light green, false positives) were mainly localized at the central pith. It should be noticed that the first slice of the frying class (top right in Figure 5) presented many more FP than TN pixels. This slice belonged to the cultivar “Agria” and such misclassification could be due to the high RS content of this cultivar with respect to the rest of the cultivars in the frying class. Moreover, the last slice of the cooking class (bottom left in Figure 5), belonging to cultivar “Rudolph,” was the worst classified in this class with a higher number of FN than TP. Cultivar “Rudolph” gave the lowest value of RS in this class, unlike cultivar “Agria.” For this reason, it is hypothesized here that the RS content may have influenced the classification of the pixels.

The potential of HSI to classify potato tubers based on different aspects has been broadly evaluated. However, this study is novel in tackling the classification of potatoes regarding their industrial aptitude by means of HSI. Most authors have focused on HSI classification of defective tubers. For instance, Ji et al. (30) combined the use of a HSI system and support vector machines (SVM) to classify 600 potatoes into six groups: Intact ones, green skin, germination, dry rot, wormhole and damage. For image acquisition, a visible-near infrared system in the range of 400–1,000 nm was used, and the mean spectra of each potato was selected for further analysis. Authors developed a linear discriminant analysis (LDA) to reduce the dimension of the data and the SVM model to classify the groups. Excellent accuracy was achieved up to 90% with SNV pre-processing. Similar results were obtained by Zhang et al. (31) while classifying potato defects, although they used a multispectral imaging system instead of a HSI. A total of 417 potato samples were used in their experiment and 25 spectral images were acquired for each tuber in the 676–952 nm spectral range. A SVM model was used to classify different defects of potato achieving an accuracy of 90.70% for the test set. Ye et al. (32) studied the detection of minor bruises in potatoes by a visible-near infrared HSI portable device covering the spectral range from 400 to 1,000 nm. They used 220 potato samples free of any damage and diseases and hit them in a controlled way by releasing a pendulum arm equipped with a ball in one end at an angle of 57°. Each tuber was impacted 3 times defining 3 bruise levels as: level I (hit one time), level II (hit twice) and level III (hit 3 times). Hyperspectral images were acquired before and 1 h after each impact. SVM models were carried out reaching accuracies up to 95% for the test set. A somehow similar study was conducted by López-Maestresalas et al. (33) with the objective of classifying potato tubers as either healthy or bruised. For this, 188 tubers were divided into two groups of the same size (healthy and damaged). The latter group was subjected to a controlled impact at the laboratory and hyperspectral images were recorded at 1, 5, 9, and 24 h after damage. Two hyperspectral systems were used, covering the 400–1,000 nm and the 1,000–2,500 nm spectral ranges. PLS-DA models were developed using the mean and the individual spectra of each tuber. Excellent classification rates were achieved with more than 98% of tubers correctly classified. At the same time, authors identified early bruises in potatoes within 5 h after bruising, with an accuracy of 97.12%.

However, the application of HSI to the study of potatoes has gone beyond damage detection. In this context, Rady et al. (34) identified sprouting activity in potatoes during storage by Vis-NIR HSI. They recorded 400 tubers by a HSI system covering the 400–1,000 m spectral range. Different machine learning techniques were tested to classify tubers as having either high or low sprouting activity. Very high classification accuracy values were obtained of 87.5 and 90% for sliced and whole samples, respectively. The same year, Xiao et al. (35) conducted a study to detect the color parameters and water content of fresh-cut potato tuber slices by HSI combined with multivariate analysis. For the experiment, 30 tuber slices of 3-mm each were taken out from 6 different tubers, resulting in 240 samples. A HSI system in the Vis-NIR region (380–1,030 nm) was used to acquire the images of the fresh-cut slices. Least Squares Support Vector Machines (LS-SVM) models were developed to predict and show the spatial distribution of color and water content in the slices. Determination coefficients of 0.84 and 0.77 were obtained in the prediction test for 5 color indicators and the water content, respectively. In a more recent study, Wang et al. (36) predicted the starch content of potatoes and visualized its distribution in fresh-cut slices by HSI. A system covering the 380–1,000 nm spectral range was used to acquire the images of 96 potato slices of 0.2 cm. Different pre-processing of data was tested along with wavelength selection methods to develop PLS regression models for starch content prediction. However, the best result was obtained using the full-spectrum pre-processed by SNV with a correlation coefficient of 0.9 in the prediction set. Regarding the visualized distribution of starch, authors found that it was mainly located along the cortical tissue with the pith having less starch content. This is in accordance with the results obtained in section “Spectral pre-processing.”

Li et al. (37) also used fresh-cut potato slices as sample material to detect *Escherichia coli* (*E. coli*) on their surface by means of HSI. For this, *E. coli* suspensions were prepared to colonize on the surface of potato slices. A total of 128 samples were prepared and analyzed by a HSI in the 400–100 nm spectral range. PLS and back-propagation neural network (BP-NN) models were established to predict *E. coli* based on full-spectrum and characteristic wavelengths. Best performance was obtained by the BP-NN model based on full-spectrum, with an overall accuracy of 97.6%. In a different study, Rady et al. (38) evaluated the sugar content in potatoes over 3 growing seasons (2008, 2009, and 2011) by HSI. The dataset was comprised of 1,210 tubers that were analyzed for glucose and sucrose content and measured in the 400–1,000 nm spectral range. PLS regression models along with PLS-DA and K-nearest neighbor (Knn) were developed to predict the sugar content and classify tubers into two classes: high or low sugar level. The best PLS model was obtained for the prediction of glucose with a correlation coefficient of cross-validation of 91.8%. Best classification accuracy was obtained for glucose levels using Knn with 91.3% of samples correctly classified in the test set.

The large number of studies in this field and the recentness of these papers demonstrate that this is a current field of research with many possibilities. However, as commented above, there is no other study focused on the HSI classification of tubers according to their industrial aptitude to the best of our knowledge. Therefore, this study can be considered novel in this field with very promising results. Even so, for future research it would be interesting to cover a series of aspects such as including a larger dataset to encompass more variability and checking the acrylamide content once the potatoes are fried to establish the correlation with RS. It would also be convenient to try segmenting the pith tissue to obtain better classifications.

## Conclusion

The capability of NIR HSI to discriminate very similar potato cultivars into two industrial aptitudes (cooking or frying as crisps) has been demonstrated in this work. We obtained very good classification accuracies up to 90% of samples correctly classified in the external validation when using the mean spectra of the whole dataset and the combination of SM+SNV+MC pre-processing. Moreover, we accomplished a pixel-wise classification to build chemical images of the samples under study. With the latter approach, accuracies above 66% were obtained in the external validation dataset using the full spectrum. Besides spectral data pre-processed with 1D+SNV+MC achieved the best classification result with an accuracy of 71.97% and an error rate of 0.279. To cope with the vast amount of data provided by the hyperspectral systems, a forward iPLS method was used to rebuild the PLS-DA models achieving accuracies above 78 and 68% in CV and validation datasets, respectively. Best classification result was obtained for spectral data pre-processed by 1D+MSC+MC using 100 wavelengths (20 intervals) with 71.86% of pixels correctly classified in the external validation dataset. The classification maps obtained showed that false negative pixels were mainly located at the edges of the fresh-cut slices while false positive were principally distributed at the central pith, which has singular characteristics. Therefore, subsequent analyses should be performed after segmentation and removal of the pith tissue.

According to the results obtained, the use of NIR HSI coupled with PLS-DA may have potential for rapid discrimination of industrial aptitude of potatoes, allowing the selection of cultivars that best suit consumer preferences.

## Data availability statement

The raw data supporting the conclusions of this article will be made available by the authors, without undue reservation.

## Author contributions

AL-M: data curation, formal analysis, investigation, methodology, supervision, validation, visualization, and writing—original draft and reviewing, and editing. GO-L: data curation, investigation, methodology, visualization, and writing—reviewing. CL-M: data curation, formal analysis, investigation, methodology, software, supervision, validation, writing—original draft and reviewing, and editing. CJ and JR: conceptualization, resources, writing—reviewing, and editing. CP-A: writing—reviewing and editing. SA: conceptualization, funding acquisition, investigation, project administration, resources, writing—reviewing, and editing. All authors contributed to the article and approved the submitted version.
