# Supplementary material for: Evaluation of near-infrared hyperspectral imaging for the assessment of potato processing aptitude
Source: Front Nutr. 2022 Oct 17;9:999877. doi: 10.3389/fnut.2022.999877 (PMC9618585; doi:10.3389/fnut.2022.999877)
Supplement: Supplementary file 1 [file Data_Sheet_1.pdf]

Supplementary Material

1 Supplementary Figures and Tables

Table S1. Characteristics of potato samples.

Cultivar	Origin[a]	Breeder Agent[a]	Shape[a]	Colour of flesh/skin[a]	Industrial aptitude
Ambition	Netherlands	Agrico UK Ltd	Long oval	Medium yellow/yellow	Cooking
Laudine	Austria	Agrico UK Ltd	Long oval	Medium yellow/yellow	Cooking
Levantina	Germany	Europlant	Oval	Light yellow/yellow	Cooking
Madeleine	Netherlands	Agrico UK Ltd	Long oval	Light beige/yellow	Cooking
Rudolph	United Kingdom	Agrico UK Ltd	Short oval	White/red	Cooking
Agria	Germany	Agrico UK Ltd	Oval to long	Yellow/white to yellow	Frying crisps
Corsica	Netherlands	Agrico UK Ltd	Short oval	Medium yellow/yellow	Frying crisps
Hermes	Austria	GB Seed Industry	Oval to round	Light yellow/White to yellow	Frying crisps
Lady Amarilla	Netherlands	Meijer Potato UK	Oval	Yellow/yellow	Frying crisps
Lyoness	Europlant	Europlant	Oval	Yellow/yellow	Frying crisps

[a] Source: European Cultivated Potato database (2021).

Table S2. Combinations of the different pre-processing used in iPLS classification including the wavelength ranges selected.

Pre-processing	Interval size	#V	λ ranges
SM+SNV+MC	5	110	994-1038, 1056-1147, 1182-1351, 1370-1383, 1417-1430
SM+MSC+MC	5	105	994-1038, 1056-1069, 1088-1147, 1182-1336, 1370-1398, 1417-1430
1D+MC	1	24	1109, 1119, 1185, 1191, 1194, 1201, 1207, 1213, 1298, 1307, 1326, 1336, 1383, 1398, 1401, 1423, 1452, 1458, 1508, 1533, 1568, 1583, 1590, 1630
1D+SNV+MC	5	95	994-1038, 1088-1100, 1119-1147, 1166-1179, 1198-1210, 1229-1304, 1323-1336, 1401-1430, 1480-1508, 1527-1540, 1668-1681
1D+MSC+MC	5	100	1009-1022, 1041-1053, 1072-1085, 1103-1163, 1182-1210, 1245-1257, 1292-1304, 1323-1336, 1386-1398, 1417-1430, 1480-1540, 1574-1587, 1621-1634

Abbreviations: V: Variables; λ ranges: Wavelength ranges selected.

1.1 Supplementary Figures

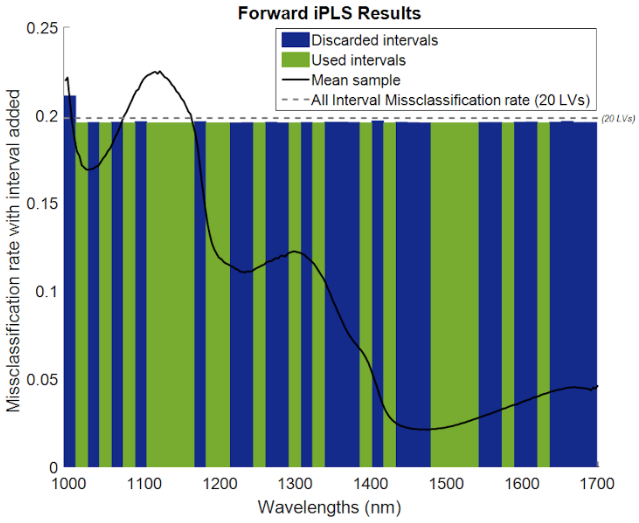

Supplementary Figure 1. Forward iPLS results obtained using after 1D+MSC+MC pre-processing
